# Motion Freeze for Respiration Motion Correction in PET/CT: A Preliminary Investigation with Lung Cancer Patient Data

**DOI:** 10.1155/2014/167491

**Published:** 2014-08-28

**Authors:** Tzung-Chi Huang, Kuei-Ting Chou, Yao-Ching Wang, Geoffrey Zhang

**Affiliations:** ^1^Department of Biomedical Imaging and Radiological Science, China Medical University, 91 Hsueh-Shih Road, Taichung City, Taiwan; ^2^Department of Biomedical Informatics, Asia University, Taichung City, Taiwan; ^3^Department of Radiation Oncology, China Medical University Hospital, Taichung City, Taiwan; ^4^Department of Radiation Oncology, Moffitt Cancer Center, Tampa, FL, USA

## Abstract

*Purpose*. Respiratory motion presents significant challenges for accurate PET/CT. It often introduces apparent increase of lesion size, reduction of measured standardized uptake value (SUV), and the mismatch in PET/CT fusion images. In this study, we developed the motion freeze method to use 100% of the counts collected by recombining the counts acquired from all phases of gated PET data into a single 3D PET data, with correction of respiration by deformable image registration. *Methods*. Six patients with diagnosis of lung cancer confirmed by oncologists were recruited. PET/CT scans were performed with Discovery STE system. The 4D PET/CT with the Varian real-time position management for respiratory motion tracking was followed by a clinical 3D PET/CT scan procedure in the static mode. Motion freeze applies the deformation matrices calculated by optical flow method to generate a single 3D effective PET image using the data from all the 4D PET phases. *Results*. The increase in SUV and decrease in tumor size with motion freeze for all lesions compared to the results from 3D and 4D was observed in the preliminary data of lung cancer patients. In addition, motion freeze substantially reduced tumor mismatch between the CT image and the corresponding PET images. *Conclusion*. Motion freeze integrating 100% of the PET counts has the potential to eliminate the influences induced by respiratory motion in PET data.

## 1. Introduction

Positron emission tomography/computed tomography (PET/CT) combining with the functional F-18 fluoro-2-deoxyglucose (^18^F-FDG) PET scan and the anatomical CT scan has become an essential modality for detecting tumors, planning radiation treatment, and evaluating response to therapy [1–3]. However, the respiratory motion during PET acquisition leads to artifacts in PET/CT. As the CT acquisition is much faster (in seconds) than PET scan (in minutes), CT image represents an almost instantaneous snapshot in comparison to the averaged PET images. Consequently, CT data is not always in spatial correspondence with the PET data and CT applied for attenuation correction (AC) in PET may lead to the underestimation of SUV_max⁡_ of tumors, overestimation of tumor volume, and mismatched PET and CT images and tumor inaccurate localization, hence potential misdiagnoses [4–8].

Several methods have been investigated mostly to manage the respiratory motion problem. These methods include breathing instruction, respiratory-gate (4D) PET/CT, motion-corrected PET reconstruction, and postprocessing methods [9–15]. Breathing instruction methods like deep-inspiration breath-hold (DIBH) during the CT scan reduce the occurrence and the severity of respiratory curvilinear artifacts on coregistered PET/CT images. A study on 108 patients showed that the SUV_max⁡_ increased as much as 51.8% on average from free-breathing PET to DIBH PET for lesions in the lower lung region as reported by Kawano et al. [9]. De Juan et al. reported that PET reconstructed images from patients of normal end-expiration breath-hold group had 28% fewer artifacts as compared with imaging by free breathing imaging [10]. These breathing instruction methods are not feasible for patients with limited pulmonary function to control their breathing. Nehmeh et al. spatially matched the 4D CT data with the gated PET images according to the externally monitored breathing signal and showed improved lesion registration [11]. Cine average CT was developed for AC in PET and showed significantly less severe misalignments and artifacts as compared with conventional helical CT (HCT) based AC [12]. The main problem of 4D technology is relatively high radiation dose compared to conventional HCT. With combining gated CT and imaging processing, the interpolated average CT used for PET/CT AC corrected the PET/CT misregistration and enhanced lesion quantitation accompanied with radiation dose reduction was proposed by Huang et al. [13, 14]. However averaging caused the lack of total effective counts in PET data. Other studies using motion estimation into the iterative reconstruction process lowered image noise of a gated PET frame but utilized lower percentage of the total PET counts [15].

Previous studies waste counts in PET images acquisition for respiratory motion reduction. In this study, we proposed the motion freeze concept to use 100% of the counts collected by recombining 100% of the counts acquired from all phases of gated PET data into a single 3D PET data, with correction of respiration by deformable image registration on the 4D CT images.

## 2. Material and Methods

### 2.1. Data Acquisition

The current study was conducted from November to December 2013. Six patients with diagnosis of lung cancer confirmed by oncologists were recruited. The summary of the clinical characteristics of the patients is shown in Table 1. The patients were all injected with 370 MBq of ^18^F-FDG. During the uptake phase that lasted for approximately 40 minutes, the patients remained in a still position. The first whole-body image was obtained when the patients were in a supine position and the acquisition time per bed position was 1.5 min. Free-breathing whole-body CT was conducted at 120 kV in helical mode (HCT) with a smart mA (range 30–210 mA), 1.375 : 1 pitch, and 0.5 s gantry rotation. For the thoracic PET, the patients were scanned for two bed positions with 1.5 min/bed. The transaxial field-of-views (FOVs) for PET and CT were set on 70 and 50 cm, respectively.

PET/CT scans were performed with Discovery STE system (GE Healthcare, Milwaukee, WI, USA). The 4D PET/CT with the Varian real-time position management (RPM system, Varian Medical Systems, Inc. Palo Alto, CA) for respiratory motion tracking was followed by a clinical 3D PET/CT-HCT scan procedure in the static mode. During all studies, the patients remained in the same position. 4D PET/CT data were acquired into 10 discrete bins in synchronization with the breathing cycle using phase gating mode [11]. 4D PET/CT protocol consisted in a 4D CT scan over the thoracic region. The 4D CT data was performed by using a step-and-shoot cine mode acquisition technique and the sinograms were reconstructed on a 512 × 512 image matrix. The pixel size in the transaxial slice of the 4D CT images was approximately 0.98 × 0.98 mm^2^, and the slice thickness was 2.5 mm. In 4D CT, the 10 respiratory phases were labeled as T5%, T15%,…, T95%, with the T5% phase corresponding approximately to the normal end-inspiration and the T55% to the end expiration. After the termination of the 4D CT scan, a 4D PET scan of two PET FOVs centered over the same anatomical region was acquired in the gated mode. 4D-PET data were corrected for attenuation with 4D CT images, then with respiratory phase matched cine CT images [16]. 4D PET images were reconstructed on a 128 × 128 image matrix with a voxel size of 4.46 × 5.46 × 3.27 mm^3^. The PET images were linearly interpolated to the resolution and geometry of the CT scan for fusion image. The ordered subset expectation maximization reconstruction method was used with 2 iterations and 20 subsets and the postreconstruction Gaussian filter was applied with a full-width at half-maximum of 7 mm. Written informed consent was obtained from all patients, and the collection of clinical patient data was approved by the ethics committees of the clinical institution.

### 2.2. Motion Freeze

**Figure 1:**
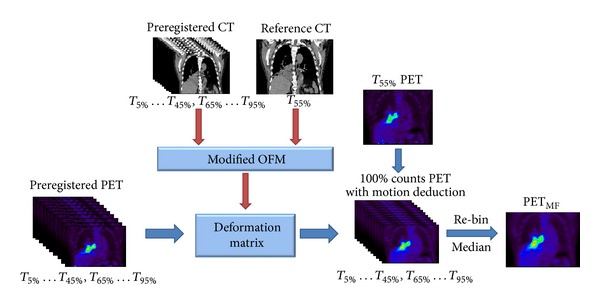
Flow diagram of the motion freeze process used in this study.

The purpose of motion freeze is to apply the deformation matrix calculated by optical flow method (OFM) [17], one of deformable image registration methods, to generate a single 3D effective PET image using the total 100% counts from different gates. Two new features including the multiloop calculation and the multiresolution to enhance the accuracy of registration were implanted into OFM [18]. The modified OFM based on image intensity gradient was utilized for deformable image registration and the accuracy of registration was reported in previous studies [18–20]. The deformable registration accuracy in the presented patient cohort is validated using manual annotation. In this study, the T55% (end expiration) phase, which is the most reproducible phase in the respiration cycle, was selected as the reference phase. The elements of deformable image registration matrix are three-dimensional vectors with magnitudes relating the computed displacement of voxels from the image of interest to the reference image. Once this deformation matrix has been calculated, the matrix can be applied to map different phases of 4D PET images to the reference phase of PET. Finally, each SUV of the corresponding voxel in the resulted 3D PET image, PET_MF_, was determined by the statistic median process, in which 100% of counts from all the phases was included. The flow diagram of the motion freeze which CT derived motion fields are used to correct PET was shown in Figure 1.

### 2.3. Quantitative Analysis

The PET data acquired from conventional static PET/HCT technology were denoted as PET_3D_. In the PET images, a 3D volume of interest (VOI) was automatically drawn. The lower threshold was set to 42% of the maximum activity concentration value within the lesion [21]. The mean SUV (SUV_mean_) and maximum SUV (SUV_max⁡_) in the VOI were calculated. The corresponding delineation for VOI in the CT images was conducted by an experienced radiation oncologist. The 4D PET phase with the highest SUV_max⁡_ (best bin) was selected as PET_4D_ for evaluation. The rebinned PET image by motion freeze approach was defined as PET_MF_. Quantitative analysis was performed by comparing the SUV_mean_, SUV_max⁡_, and tumor volume and the percentage difference (PD) was calculated using PET_3D_ as reference. The coordinates of the centroid of the lesion displayed in the PET and corresponding CT images were determined based on the delineated VOIs. The distance “*d*” between the lesion centroid identified using PET and the one in the associated CT image was then measured. Tumor deformation (TD) between the end-inspiration (T5%) and the end-inspiration (T55%) from 4D-CT data was also calculated using the average of displacements in three directions: the anterior-posterior (AP), lateral (LAT), and superior-inferior (SI) [22].

## 3. Results

**Figure 2:**
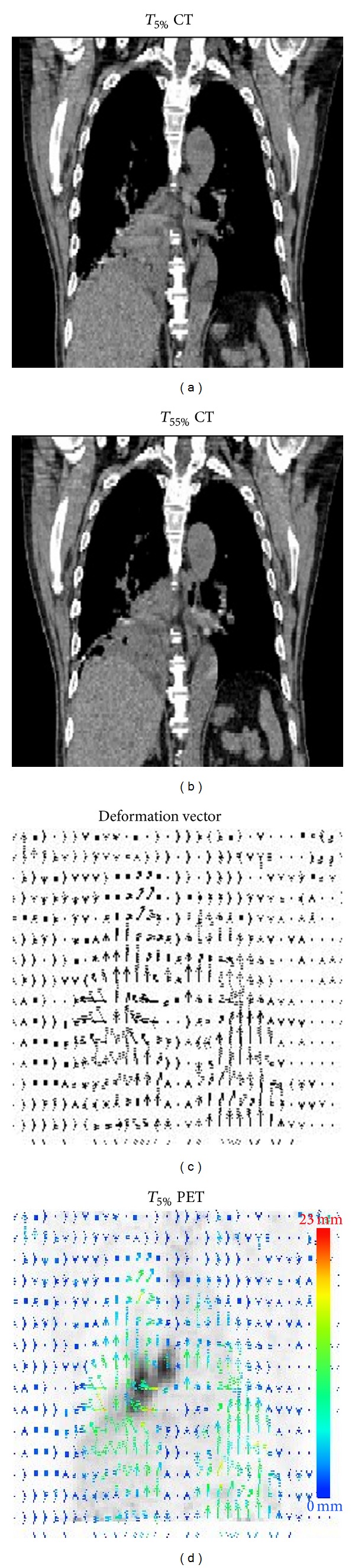
Example of deformable image registration using the 3D optical flow method to map the T5% PET to T55% PET. (a) A coronal view of the normal end-inspiration CT (T5%, original image) of 4D-CT. (b) The corresponding slice of the location in (a) at end-expiration CT (T55%, reference image) of 4D CT. (c) The calculated deformation vectors for the registration of original image to the reference. (d) The corresponding T5% PET with the 3D OFM deformation vectors superimposed.

**Figure 3:**
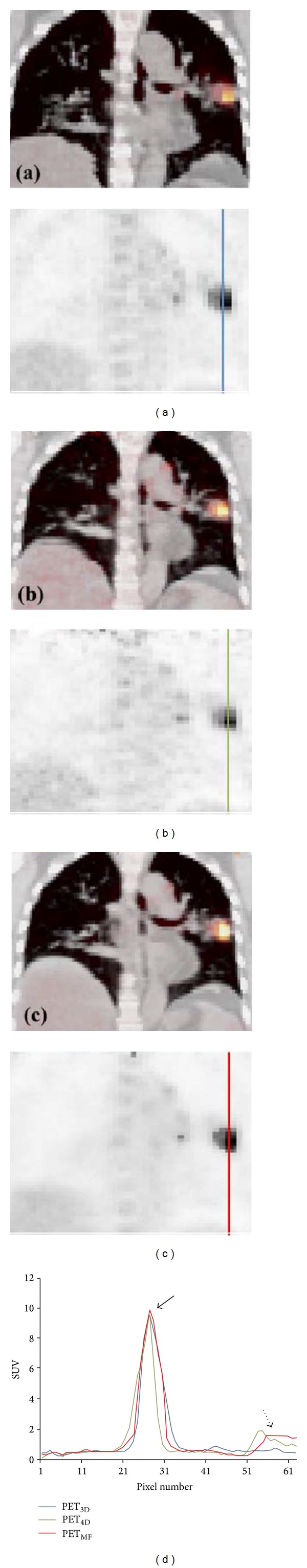
The coronal views of the fused PET/CT images from (a) 3D, (b) 4D (best bin), (c) motion freeze technology for patient number 4. A lesion in the left upper lobe of the lung and artifacts around the right diaphragm were observed on the 3D HCT images, and (d) vertical image profiles drawn across the lesion on PET_3D_, PET_4D_, and PET_MF_ images. Black arrow indicated the lesion area and the dotted black arrow indicated the diaphragm region.

An example (Patient number 1) of deformable image registration using the 3D optical flow method to map the T5% PET to T55% PET is shown in Figure 2. For Patient number 4, as demonstrated in Figure 3, the lesion in the left upper lobe was substantially improved for PET_4D_ and PET_MF_ as compared to PET_3D_, with increase of SUV_mean_ of 27% and 81%, respectively. The identification of the PET images was also improved for PET_MF_ as compared to PET_3D_ in this patient as indicated in the line profiles drawn on Figure 3(d), where the blurring image appeared to be smaller with improved structure details in the outside of lesion on PET_MF_. The PET_3D_ had severe cold artifacts around the left diaphragm region, while the artifacts were reduced in the PET_MF_.

The SUV_mean_, SUV_max⁡_, and tumor volume for all patients are summarized in Table 2. Compared to PET_3D_ and PET_4D_, the SUV_mean_ in PET_MF_ was higher for all the tumors. The average increase of PDs of SUV_mean_ for all patients was 14% and 22% for PET_4D_ and PET_MF_ as compared to PET_3D_, respectively. The tumor volumes measured in PET_MF_ were significantly smaller than the values measured in PET_3D_ and PET_4D_. The PDs of the tumor volume measured in PET_MF_ compared to PET_3D_ were in the range between 17% and 19%. The values of SUV_max⁡_ were all increased by PET_MF_ as compared of PET_3D_.

Estimates of TD between the normal end-inspiration and end-expiration phases and the differences in the centroid of the tumors (*d*) are represented in Table 3. PET_MF_/CT_T55%_ fusion image substantially reduced tumor mismatch *d* between the CT image and the corresponding PET images, as shown in Table 3, with an average decrease of 6.1 mm among all of the lesions as compared to 3D-PET/HCT, whereas an average decrease of 4.3 mm was observed when using 4D-PET/CT (best-bin).

## 4. Discussion

The idea of motion freeze is conceived by GE Q. Freeze technology (GE Healthcare, Milwaukee, WI, USA) is the latest commercial product coping with motion correction for PET/CT. Q. Freeze technology registering PET by OFM was applied for motion estimation, whereas the validation with ground truth was missing and only the relative correlation coefficient was presented [23]. The OFM basically calculates the flow information according to the changes in image intensity between two images. The image resolution is generally coarse in PET image and also the structure information was not represented in PET image. Consequently, the precise registration in PET image was difficult to achieve. The 4D CT images were used for registration using OFM in this study. The validations of the proposed OFM were reported in previous phantom and clinical patient studies [18–20]. In OFM for deformable image registration, CT as image modality is superior to PET. Although model tissue viscosity and elasticity are used to take into consideration the anatomical environment after OFM registration in GE Q.Freeze technology, the model should not be identical for all patients and usually not feasible for patients with lung injuries [24].

Respiratory motion will blur the activity image and hence reduce the measured activity concentration and corresponding SUV. Motion freeze registers all the gated phases to the selected gated phase (T55%) and the 100% counts are all used to provide the best quality of PET image with motion deduction. The quantitation recovery on both the SUV derived from the motion freeze process as compared with the SUV obtained from the corresponding static PET was observed in our results. The visual blur in the static image resulting from respiratory motion generally appears stretched along the direction of the motion and can be quantified by the tumor volume. The estimate of the tumor volume obtained from a static image is usually higher than that for individual gates. The blur reduction was observed by the comparison between the tumor volumes obtained from the motion freeze processing and that from the corresponding static (3D) image.

The improvement of SUV_max⁡_ in 4D-PET and PET_MF_ was not improved substantially as compared to 3D-PET in Patient 4. In this case, the tumor was in the left upper lobe (Table 1) thus the respiratory motion was relative small (Table 3). Because of the small motion, the blurring effect on SUV_max⁡_ in 3D PET is relatively smaller. Besides, the 3D PET was obtained 21 minutes later than the 4D PET and thus the uptake in the tumor could be larger at the time when the 3D PET was taken. Similar cases were reported in another study [22]. Even in the case with little improvement of SUV_max⁡_ represented in 4D PET, motion freeze can improve the quantification of counts for such the case too. After this feasibility study, we may conduct a detailed study with more patients' data to quantitatively investigate how the motion size affects the SUV_max⁡_ using this method.

Because the motion freeze resulted from 4D PET/CT imaging, different gating techniques or schemes in 4D PET/CT imaging could affect the motion freeze outcomes [16, 25, 26]. The effects of the gating schemes from 4D-CT for AC applied on PET_MF_ effectiveness are being further investigated in our ongoing study.

The major limitations to widespread clinical adoption of respiratory motion correction are the need to use extra CT dose and the need for additional acquisition time. Motion freeze technology combining the interpolated CT technology has high potential to overcome these challenges by using the entire acquired data to create a single 3D motion corrected image and to provide quantitative accuracy equivalent to 4D PET/CT with shorter acquisition times and less dose reduction [13, 14]. Unlike conventional 4D PET imaging, motion freeze technology combines 100% of the PET counts into a 3D motion corrected image that has a comparable acquisition time and the equivalent image noise of a static acquisition. With aids of the motion freeze technology, the acquisition of each bed could decrease and still keep the high counts for PET image. In radiation dose reduction from the 4D-CT, the equivalent 4D phase-matched PET/CT could be done by incorporation of active breathing controller and the interpolated CT (ICT) method used for PET/CT attenuation correction mismatch [27]. The resulting image has advantages in frozen respiratory motion, increased SUV, less acquisition time, and CT dose reduction.

For patients who have serious irregular breathing amplitude/pattern which may cause breathing pattern change between CT and PET scans, this method may suffer some uncertainty. In 4D PET/CT, 4D-CT is often used for PET image attenuation correction. For patients who cannot regularly breathe, artifacts are likely in the reconstructed 4D-CT. If breathing amplitude/pattern is changed between CT and PET scans, the 4D-PET data would not be accurate after attenuation correction. Any postscan image processing would be consequently affected. After the CT deformation matrices, which are possibly generated by a different breathing amplitude, are applied to the PET data, the integrated PET may not be exactly reflecting the reality.

## 5. Conclusion

In this study, the motion freeze technology was developed and PET acquisitions were corrected for respiratory motion, leading to superior image quality and increased quantitative SUV in comparison to conventional static imaging. Motion freeze also reduced the lesion volume and lesion maximum activity errors compared to conventional static PET imaging. Motion freeze integrating 100% of the PET counts has the potential to eliminate the influences induced by respiratory motion in PET data.

## Figures and Tables

**Table 1:** Clinical characteristics of study subjects and tumors.

Patient #	Sex	Age (y)	Histology	Location	Type	Stage
1	M	64	NSCLC	Right lower lobe	Lymph node	III
2	M	59	NSCLC	Left lower lobe	Lymph node	III
3	M	63	NSCLC	Right lower lobe	Lymph node	III
4	F	57	NSCLC	Left upper lobe	Lymph node	III
5	M	61	NSCLC	Right lower lobe	Lymph node	III
6	M	64	NSCLC	Left lower lobe	Lymph node	III

*NSCLC = Non-small-cell lung carcinoma.

**Table 2:** Summary of different quantitative results for 3D, 4D, and motion freeze methods.

Patient number	SUV_mean_			SUV_max_			Tumor Volume (cm^3^)		
	PET_3D_	PET_4D_	PET_MF_	PET_3D_	PET_4D_	PET_MF_	PET_3D_	PET_4D_	PET_MF_
1	7.4	7.5	7.6	13.5	13.7	13.9	68.2	51.1	50.2
2	7.7	7.9	7.9	13.2	15.3	15.8	173.1	144.7	140.0
3	6.6	8.2	8.4	12.4	14.8	16.3	120.0	109.4	100.8
4	2.6	3.3	4.7	9.9	9.9	10.0	22.0	14.7	12.7
5	1.8	1.8	2.1	8.2	9.3	9.8	132.2	128.0	123.1
6	3.0	3.8	4.4	14.8	15.9	15.9	42.2	34.8	27.6

**Table 3:** Summary of tumor motion estimations and differences of centroid of the tumors.

Patient number	Deformation of tumor motion (mm)				*d* (mm)		
	AP	LAT	SI	TD	3D-PET/HCT	4D-PET/CT	PET_MF_/CT_T55%_
1	1.1 ± 1.0	0.9 ± 0.9	7.3 ± 2.6	7.7 ± 2.6	22.7	16.4	11.4
2	1.9 ± 1.2	1.4 ± 1.0	3.1 ± 1.4	4.4 ± 1.1	6.1	5.5	4.6
3	0.9 ± 1.2	0.5 ± 0.5	2.5 ± 1.9	2.88 ± 2.3	15.5	10.5	10.4
4	0.6 ± 0.5	0.9 ± 1	2.6 ± 1	3.0 ± 0.8	7.1	3.1	1.4
5	3.2 ± 2.2	2.5 ± 2.2	3.6 ± 1.9	5.9 ± 2.6	13.5	6.1	5.7
6	3.6 ± 2.5	1.2 ± 0.8	8.5 ± 5.4	9.6 ± 2.1	7.7	5.2	2.3
